# Transgene-Free Direct Osteogenic Reprogramming Using Cell-Permeable Octamer-Binding Transcription Factor 4/Core-Binding Factor β Fusion Proteins

**DOI:** 10.34133/bmr.0320

**Published:** 2026-02-03

**Authors:** Manho Kim, Jaeyoung Lee, Wijin Kim, Songrae Kim, Jongmin Park, Ju Hyun Park

**Affiliations:** ^1^ Department of Biomedical Science, Kangwon National University, Chuncheon, Republic of Korea.; ^2^ Metropolitan Seoul Center, Korea Basic Science Institute (KBSI), Seoul, Republic of Korea.; ^3^ Department of Chemistry, Kangwon National University, Chuncheon, Republic of Korea.; ^4^ Institute of Molecular Science and Fusion Technology, Kangwon National University, Chuncheon, Republic of Korea.

## Abstract

Bone-related disorders, including fractures and osteoporosis, remain substantial clinical challenges, partly because of the limited availability of reliable osteogenic cell sources and complications associated with current therapies. To address these limitations, this study introduces a novel protein-based direct reprogramming platform for the conversion of human dermal fibroblasts into functional osteoblasts using only 2 transcription factors, octamer-binding transcription factor 4 (Oct4) and core-binding factor β (Cbfβ), fused to the silkworm-derived cell-penetrating protein, 30Kc19. Genetic fusion with 30Kc19 markedly improves the stability and cellular uptake of both Oct4 and Cbfβ, resulting in recombinant constructs (Oct4-30Kc19 and Cbfβ-30Kc19) that achieve high reprogramming efficiency with negligible cytotoxicity, outperforming plasmid DNA-based methods. The protein-induced osteoblasts (piOBs) exhibit a characteristic osteoblast morphology, express established osteogenic markers, and display a global transcriptomic profile that aligns with key features of primary human osteoblasts. Importantly, transplantation of piOBs into a murine calvarial defect model induces substantial new bone formation, demonstrating in vivo therapeutic efficacy. By leveraging the unique cell-permeable and protein-stabilizing properties of 30Kc19, this streamlined 2-factor system represents a potentially safer, more scalable, and clinically feasible strategy for regenerative therapies targeting bone diseases, circumventing the inherent risks associated with viral vectors and genomic integration.

## Introduction

Bone provides the structural framework of the body and enables movement [1]. Bone-related disorders, including osteoporosis and fractures, substantially diminish quality of life, particularly in older adults in whom fractures are associated with increased mortality [2]. Current therapeutic strategies for bone-related disorders include autologous and allogeneic bone grafting, gene therapy, and cell-based interventions [3]. Although autologous bone grafting is considered the gold standard owing to its combined osteoinductive, osteoconductive, and osteogenic properties, it has significant morbidity and a high complication rate, frequently resulting in patient rejection [4]. Cell-based therapies are promising alternatives owing to their inherent osteogenic potential [5]. However, ethical concerns, production costs, and limitations in cell availability and scalability continue to hinder their widespread clinical implementation.

Among cell-fate engineering approaches, induced pluripotent stem cell (iPSC) technology has revolutionized regenerative medicine by enabling the generation of patient-specific pluripotent stem cells [6]. However, the requirement to transit through a pluripotent intermediate state introduces additional complexity, prolongs the temporal requirement for therapeutic cell production, escalates costs, and elevates the risk of tumorigenesis. In contrast, direct reprogramming strategies bypass this pluripotent stage, thereby abbreviating the reprogramming timeline and reducing oncogenic potential [7]. Furthermore, in vivo direct reprogramming, a strategy involving the direct administration of reprogramming factors to endogenous tissues, holds the potential to generate functional cells in situ, obviating the need for subsequent cell transplantation procedures. Despite these advancements, safe and efficient factor delivery remains a central barrier to both in vitro and in vivo applications.

Most reprogramming approaches rely on gene delivery vehicles, such as viral vectors (e.g., retrovirus or lentivirus), plasmid DNA, mRNA, or small-molecule cocktails. While viral vectors achieve high gene expression efficiency, they present inherent risks of insertional mutagenesis and subsequent tumorigenesis, limiting their suitability for therapeutic applications. Nonintegrating platforms, including episomal plasmids, RNA-based platforms, or nonintegrating viral vectors, partially address these safety concerns, but often yield suboptimal transgene expression and transient availability of reprogramming factors [8]. Furthermore, cells generated via direct conversion typically exhibit limited proliferative potential in contrast to iPSCs, which possess unlimited self-renewal capacity. Accordingly, therapeutic efficacy will depend on generating sufficient target cells by enhancing reprogramming efficiency while employing genome-safe delivery methods.

Protein-based reprogramming provides an intrinsically nonintegrative alternative that avoids genome modification. However, practical applications have been limited by suboptimal soluble expression, rapid proteolytic degradation, and inefficient cellular uptake. To improve intracellular delivery, diverse cell-penetrating peptides (CPPs), including TAT, Antp, and poly-arginine, have been used to translocate recombinant proteins into cells [9]. Although CPPs enhance cellular uptake, they generally do not address upstream bottlenecks in protein yield, solubility, or intracellular stability; thus, overall reprogramming efficiency has remained modest.

To overcome these limitations, we developed a fusion-protein strategy using 30Kc19, a hemolymph protein from *Bombyx mori* [10]. The 30Kc19 protein has been demonstrated to act as a multifunctional fusion partner, simultaneously enhancing cellular uptake, soluble expression, and intracellular stability of fused cargo proteins [11,12]. Notably, although 30Kc19 is larger than conventional CPPs, it maintained the transcriptional activity of fused reprogramming factors, a critical feature for successful reprogramming [12,13]. We therefore fused candidate reprogramming factors to 30Kc19, enabling the genome-safe and efficient delivery of functional factors while circumventing the key obstacles of protein-only reprogramming, including inefficient cellular uptake, poor soluble expression, and low intracellular half-life.

Our platform targets 2 pivotal inducers of osteogenesis, octamer-binding transcription factor 4 (Oct4) and core-binding factor β (Cbfβ). Oct4 is a master transcriptional regulator that is critical for maintaining pluripotency and has been widely applied for cellular reprogramming to induce pluripotency in somatic cells [14,15]. In addition to its fundamental role in pluripotency induction, Oct4 has been explored for its ability to facilitate direct cell-fate conversion into alternative lineages when combined with other lineage-specifying factors [16–18]. This approach enables terminally differentiated cells to enter an epigenetically “plastic state”, characterized by the loss of their original cell identity and the activation of lineage-specific developmental programs [17–19]. Previous studies have demonstrated that Oct4 overexpression is indispensable for the direct conversion of human fibroblasts into osteoblast-like cells [20]. Cbfβ, a non-DNA-binding cofactor of the Runx transcription factor family, is essential for osteoblast differentiation and bone matrix deposition [21,22]. We have previously shown that exogenous delivery of recombinant Cbfβ protein to human bone marrow-derived mesenchymal stem cells initiates osteogenic gene expression and promotes mineralization [23].

**Fig. 1.**
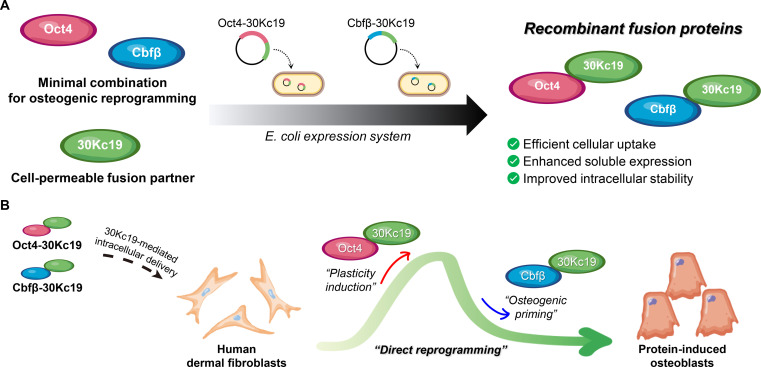
Schematic illustration of the protein-based platform for the direct osteogenic reprogramming of human dermal fibroblasts (HDFs). (A) Production of recombinant Oct4-30Kc19 and Cbfβ-30Kc19 fusion proteins. Expression plasmids encoding reprogramming factors fused with the 30Kc19 moiety were introduced into an *E. coli* expression system to generate cell-permeable recombinant proteins. (B) Direct osteogenic reprogramming via intracellular protein transduction. The fusion proteins are intracellularly delivered through 30Kc19-mediated transport. Oct4-30Kc19 induces a state of cellular plasticity, while Cbfβ-30Kc19 promotes osteogenic lineage commitment, synergistically driving the conversion of HDFs into functional osteoblasts.

Based on these observations, we hypothesized that a dual-factor combination approach, leveraging Oct4 to induce a plastic state in somatic cells and Cbfβ to direct osteogenic lineage commitment, would synergistically enhance the efficiency and fidelity of osteoblast reprogramming. In this study, we report the design, production, and functional evaluation of Oct4-30Kc19 and Cbfβ-30Kc19 fusion proteins in the direct conversion of human dermal fibroblasts (HDFs) into functional osteoblasts (Fig. 1). Our findings establish a protein-based reprogramming platform that achieves genome integration-free delivery with improved safety and translational potential.

## Materials and Methods

### Plasmid construction

The pCXLE-hOct4 episomal plasmid (#27076) was obtained from Addgene (Watertown, MA, USA). To construct an episomal plasmid encoding Cbfβ, the cDNA sequence of Cbfβ was amplified using polymerase chain reaction (PCR) from the pET-23a/Cbfβ-30Kc19 plasmid. The resulting PCR product was inserted into the pCXLE-hOct4 backbone, and the Oct4 sequence was replaced with an EcoRI restriction site. The resulting plasmid was designated as pCXLE-Cbfβ. The pET-23a/Oct4-30Kc19 and pET-23a/Cbfβ-30Kc19 plasmids were constructed as previously reported [13,23]. The 30Kc19 sequence was fused to the C terminus of each reprogramming factor and cloned between the BamHI and XhoI restriction sites.

### Cell culture

HDFs were obtained from Thermo Fisher Scientific (Waltham, MA, USA) and cultured in Dulbecco’s modified Eagle’s medium (DMEM; Welgene, Daejeon, Republic of Korea) supplemented with 10% fetal bovine serum (Welgene) and 1% penicillin–streptomycin (Thermo Fisher Scientific). The medium was replaced every 2 d, and cells were passaged using trypsin–EDTA (Thermo Fisher Scientific) when confluency reached 70% to 80%. All experiments utilized HDFs at a low passage number (*p* < 5) to ensure consistent cellular characteristics. Osteogenic reprogramming was induced using osteogenic medium (OM), consisting of DMEM supplemented with 10 mM β-glycerol phosphate (Sigma-Aldrich, St. Louis, MO, USA), 50 μg/ml ascorbic acid (Sigma-Aldrich), and 100 nM dexamethasone (Sigma-Aldrich). Primary human osteoblasts (hOBs; PromoCell, Heidelberg, Germany) were cultured in Osteoblast Growth Medium (PromoCell) according to the manufacturer’s instructions. The medium was replaced every 2 d. All experiments were performed using cells at a low passage number (*p* < 4). Both cell types were maintained at 37 °C in a humidified incubator with 5% CO_2_.

### Episomal plasmid transfection

Episomal plasmids were delivered to HDFs via either polymeric transfection or electroporation. For polymeric transfection, TransIT-X2 Dynamic Delivery System (Mirus Bio, Madison, WI, USA) was used according to the manufacturer’s protocol. Briefly, transfection complexes were prepared by mixing Opti-MEM (Thermo Fisher Scientific), plasmid DNA, and TransIT-X2 reagent at a ratio of 100 μl:1 μg DNA:3 μl. Following 15-min incubation, the complexes were added to the cells, and the cultures were maintained for 24 h.

Electroporation was conducted using the Neon Transfection System (Thermo Fisher Scientific) with the 100-μl electroporation kit. For each reaction, 3 μg of episomal plasmid DNA (Oct4, Cbfβ, or both) was introduced into 6 × 10^5^ HDFs. The cells were resuspended in 100 μl of Neon Buffer R (Thermo Fisher Scientific) premixed with the plasmid DNA and subjected to 3 pulses at 1,650 V with 10-ms pulse width.

### In vitro osteogenic differentiation assay

Osteogenic differentiation was assessed by alkaline phosphatase (ALP) staining, Alizarin Red S (ARS) staining, OsteoImage Mineralization Assay (Lonza, Basel, Switzerland), and von Kossa staining (Abcam, Cambridge, UK). For ALP staining, cells were fixed with 4% paraformaldehyde (PFA) for 15 min on day 14 of reprogramming and then stained using the StemAb Alkaline Phosphatase Staining Kit II (Reprocell, Yokohama, Japan) according to the manufacturer’s protocol. For ARS staining, cells were fixed on day 24 and incubated with a 2% ARS solution for 10 min. Excess dye was rinsed with distilled water, and the bound ARS was eluted with 10% acetic acid. The eluate was then neutralized with 10% ammonium hydroxide and quantified by measuring absorbance at 405 nm on a microplate reader (Multiskan GO, Thermo Fisher Scientific) following previously reported protocols [23]. The OsteoImage Mineralization Assay and von Kossa staining were also performed on day 24. Fixed cells were processed according to the respective manufacturer’s instructions to visualize mineralized calcium deposits. Calcium accumulation was observed under a fluorescence microscope (Leica, Bensheim, Germany), and quantitative analysis was performed using ImageJ software (National Institutes of Health, Bethesda, MD, USA).

### Immunostaining

Cells were fixed with 4% PFA at room temperature for 30 min, followed by 3 washes with phosphate-buffered saline (PBS). For membrane permeabilization, the cells were incubated in PBS containing 0.25% Triton X-100 for 30 min and then blocked with 3% bovine serum albumin (BSA) in PBS containing 0.1% Tween 20 (PBS-T) for 1 h at room temperature. After washing 3 times with PBS-T, cells were incubated overnight at 4 °C with primary antibodies diluted 1:500 in 1% BSA/PBS-T. The primary antibodies used were Oct4 (Abclonal, Woburn, MA, USA), Cbfβ (Santa Cruz Biotechnology, Santa Cruz, CA, USA), osteocalcin (OCN) (Santa Cruz Biotechnology), and osteopontin (OPN) (Thermo Fisher Scientific). The next day, the cells were washed with PBS-T and incubated with secondary antibodies (Thermo Fisher Scientific) at a 1:1,000 dilution in 1% BSA/PBS-T for 1 h at room temperature. Nuclei were counterstained with 1 μg/ml 4′,6-diamidino-2-phenylindole (DAPI; Sigma-Aldrich) for 1 min. Stained cells were then visualized using a fluorescence microscope or confocal laser scanning microscope (CLSM; LSM880, Carl Zeiss, Jena, Germany).

### Recombinant protein expression and purification

Recombinant Oct4-30Kc19 and Cbfβ-30Kc19 proteins were produced using an *Escherichia coli* expression system. The BL21 (DE3) *E. coli* strain (Novagen, Madison, WI, USA), transformed with each recombinant vector, was inoculated into Luria–Bertani (LB) broth and cultured at 37 °C with agitation at 200 rpm. When the cultures reached exponential growth phase (OD_600_ 0.4 to 0.6), protein expression was induced by adding isopropyl-β-d-thiogalactopyranoside (IPTG) to a final concentration of 1 mM. After induction, the cultures were incubated at 25 °C with agitation at 200 rpm for 16 h. Then, the cells were harvested by centrifugation at 8,000 rpm for 20 min, then resuspended in His-binding buffer (20 mM tris-HCl, 0.5 M NaCl, 20 mM imidazole, pH 8.0), and lysed by ultrasonication. The lysate was centrifuged at 12,000 rpm for 30 min to collect the soluble protein fraction. For purification, the soluble fraction was loaded onto a HisTrap HP column (Cytiva, Marlborough, MA, USA) using an AKTA purification system (Cytiva). Unbound proteins were removed using His-washing buffer (20 mM tris-HCl, 0.5 M NaCl, 50 mM imidazole, pH 8.0). Finally, the target proteins were eluted with His-elution buffer (20 mM tris-HCl, 0.5 M NaCl, 350 mM imidazole, pH 8.0). The eluted proteins were buffer-exchanged into DMEM using a desalting column (Cytiva) and stored at −80 °C until use.

### Characterization of recombinant proteins

The identity of the recombinant proteins weas confirmed by Coomassie Brilliant Blue staining and Western blotting. For Coomassie staining, the purified proteins were prepared in 5× sodium dodecyl sulfate (SDS) sample buffer (LPS solution, Daejeon, Republic of Korea), boiled at 95 °C for 5 min, and separated by 10% SDS-polyacrylamide gel electrophoresis (PAGE). The protein bands were then visualized with Coomassie Brilliant Blue R-250 (Sigma-Aldrich). For Western blotting, proteins were transferred from SDS-PAGE gel onto a polyvinylidene difluoride membrane (Cytiva). The membrane was blocked for 1 h at room temperature with 5% skim milk in tris-buffered saline containing 0.1% Tween-20 (TBS-T), followed by 3 washes with TBS-T. The membrane was then incubated overnight at 4 °C with primary antibodies against Oct4 (Abclonal) or Cbfβ (Santa Cruz Biotechnology), diluted 1:500 in 1% skim milk/TBS-T. After 3 washes with TBS-T, the membrane was incubated with horseradish peroxidase-conjugated secondary antibodies (Thermo Fisher Scientific) at a 1:1,000 dilution in 1% skim milk/TBS-T for 1 h at room temperature. Finally, protein bands were detected using ECL Prime Western Blotting Detection Reagent (Cytiva).

### Cytotoxicity assay

To evaluate the cytotoxicity of the recombinant proteins, HDFs were seeded at a density of 1 × 10^4^ cells/cm^2^ in 24-well plates (SPL, Pocheon, Republic of Korea) and treated with Oct4-30Kc19 (80 μg/ml) and Cbfβ-30Kc19 (300 μg/ml). Protein treatments were applied up to 4 times depending on the experimental schedule. After the designated incubation period, cell viability was assessed using the trypan blue exclusion assay. Viable and nonviable cells were counted using a hemocytometer after staining the cells with trypan blue. For the Live/Dead assay, cells were washed 3 times with PBS and incubated for 20 min at 37 °C with 2 μM calcein-AM and 4.5 μM propidium iodide in the dark. The stained cells were visualized and imaged immediately using a fluorescence microscope (Leica).

### Recombinant protein treatment

For the evaluation of protein penetration and intracellular degradation kinetics, HDFs were seeded at a density of 1 × 10^4^ cells/cm^2^ on cell culture slides (SPL) and incubated for 24 h prior to recombinant protein treatment. Following this period, Oct4-30Kc19 (80 μg/ml) and Cbfβ-30Kc19 (300 μg/ml) were added to the culture medium. Protein uptake was assessed at 30 min, 1 h, and 8 h post-treatment. To evaluate intracellular degradation, the medium was replaced with fresh protein-free medium at the 8-h time point. Cells were then incubated for an additional 8, 16, 24, or 48 h before being subjected to immunostaining to quantify residual intracellular protein. For direct reprogramming, HDFs were treated with OM supplemented with 80 μg/ml of Oct4-30Kc19 and 300 μg/ml of Cbfβ-30Kc19. This protein treatment cycle was applied for 24 h and repeated for a total of 8 cycles. After the final treatment, the medium was changed every 2 d until day 24.

### RNA isolation and QuantSeq 3′ mRNA sequencing

QuantSeq 3′ mRNA sequencing was performed on 3 experimental groups: control (HDFs), treatment [protein-induced osteoblasts (piOBs)], and positive control (hOBs). Total RNA was isolated from cells cultured for 24 d and used for library preparation with the QuantSeq 3′ mRNA-Seq Library Prep Kit (Lexogen, Vienna, Austria), according to the manufacturer’s instructions. Sequencing was conducted on the Illumina NextSeq 500 platform (Illumina, San Diego, CA, USA). Sequencing reads were aligned to the human reference genome using Bowtie2, and transcript abundance and differential expression were analyzed using EdgeR within the R/Bioconductor environment [24]. Genes with a fold change ≥ 2 were considered significantly differentially expressed. Read-count processing and data visualization were performed using ExDEGA software v5.1.1 (eBiogen, Seoul, Republic of Korea). Gene Ontology (GO) enrichment analysis of the selected differentially expressed genes (DEGs) was performed with DAVID, and ossification-related GO terms were retrieved from QuickGO (https://www.ebi.ac.uk/QuickGO). Expression patterns of selected genes were compared among groups, and one representative GO term was further analyzed using STRING (https://string-db.org) to visualize the corresponding protein–protein interaction network.

### Gelatin cryogel fabrication and characterization

Type A gelatin (Sigma-Aldrich) was dissolved in distilled water at a concentration of 1% (w/v) and stored at 4 °C until use. For cross-linking, 50 mM 1-ethyl-3-(3-(dimethylaminopropyl) carbodiimide (EDC; Sigma-Aldrich) and 20 mM N-hydroxysulfosuccinimide (NHS; Sigma-Aldrich) were added to the cold gelatin solution. The mixture was then transferred to a prechilled mold and incubated at −20 °C overnight to induce cryogelation. Following the freezing step, the samples were lyophilized to remove ice crystals and generate a macroporous structure. The resulting cryogels were rehydrated in PBS and sterilized by ultraviolet irradiation before use.

The freeze-dried cryogel was characterized to confirm its porous architecture. It was sputter-coated with platinum and visualized using scanning electron microscopy (SEM; JSM-7900F, JEOL, Tokyo, Japan). The interconnected porosity was quantified using a water-wicking technique, and the swelling ratio was determined by comparing the freeze-dried and swollen weights. The crosslinking degree was evaluated using a ninhydrin assay. Briefly, the crosslinked or noncrosslinked cryogels were treated with a ninhydrin solution (TCI, Tokyo, Japan) and incubated in boiling water for 20 min. The absorbance of the resulting solution was measured at 570 nm using a microplate reader.

To assess the biocompatibility, 1 × 10^4^ HDFs were seeded onto each cylindrical cryogel (3.5 mm diameter × 2 mm height). Cell viability was evaluated at designated time points using a water-soluble tetrazolium salt-8 (WST-8) assay. In addition, a Live/Dead assay was performed on day 7 to assess the cytotoxicity and cell survival within the scaffold.

### In vivo bone regeneration

All animal experiments were approved by the Institutional Animal Care and Use Committee (IACUC) of Kangwon National University (Approval No. KW-210629-1). The in vivo bone regenerative capacity of piOBs was evaluated using male BALB/c nude mice (8 weeks old). The mice were housed in humidity- and temperature-controlled environments and randomly assigned to either the control (HDFs) or treatment (piOBs) group. For the calvarial defect model, the animals were anesthetized by isoflurane inhalation, and the sagittal crest was surgically exposed. A critical-sized defect (4 mm diameter) was created using a trephine bur on a dental handpiece (SAESHIN, Daegu, Republic of Korea) with sterile saline irrigation to minimize thermal injury.

Gelatin cryogels were seeded with HDFs that had been pretreated with Oct4-30Kc19 and Cbfβ-30Kc19 for 8 consecutive cycles. The cell seeding density was 5 × 10^5^ cells per scaffold. Identically prepared cryogels seeded with untreated HDFs at the same density served as controls. The cell-laden cryogels were then implanted into calvarial defects, and the surgical site was closed using nonabsorbable silk sutures. Mice were euthanized at 8 weeks post-implantation, and cranial tissues were harvested for subsequent micro-computed tomography (micro-CT) and histological analysis.

### Micro-CT

To quantify calvarial bone regeneration, harvested mouse cranial tissues were scanned using a Quantum GX2 micro-CT system (PerkinElmer, Waltham, MA, USA) at 60 kV and 6.5 W, with an exposure time of 0.5 s. Projection data were reconstructed using Analyze 11.0 software (AnalyzeDirect, Overland Park, KS, USA). The regenerated bone volume fraction (BV/TV) was calculated by determining the newly formed bone within a defined volume of interest, and trabecular separation was measured based on standard 3-dimensional (3D) morphometric parameters [25].

### Histological analysis

Histological evaluation was performed using hematoxylin and eosin (H&E) and Masson’s trichrome (MTC) staining. Harvested cranial tissues were fixed in 10% neutral-buffered formalin for 24 h and decalcified in 0.5 M EDTA (Santa Cruz Biotechnology) for 14 d. Following decalcification, the samples were equilibrated in a 30% sucrose solution for 24 h and embedded in Tissue-Tek O.C.T. compound (Sakura Finetek, Torrance, CA, USA) using aluminum molds. Frozen blocks were prepared by immersion in liquid nitrogen, and tissue sections (5-μm thickness) were obtained using a cryostat. Sections were stained with H&E, and MTC staining was performed using a commercial staining kit (Newcomer Supply, Middleton, WI, USA) according to the manufacturer’s instructions. Stained sections were imaged under optical microscopy (Leica).

For immunostaining of cryosections, sections were permeabilized with 0.25% Triton X-100 in PBS for 15 min, blocked with 3% BSA in PBS-T for 1 h at room temperature, and incubated overnight at 4 °C with primary antibodies against OCN and OPN diluted 1:500 in 1% BSA/PBS-T. After 3 washes with PBS-T, secondary antibodies (Thermo Fisher Scientific; 1:1,000) were applied for 1 h at room temperature. Nuclei were counterstained with DAPI (1 μg/ml), and images were acquired by fluorescence microscopy.

### Statistical analysis

All statistical analyses were performed using GraphPad Prism 9.2 (GraphPad Software, San Diego, CA, USA). Comparisons between 2 groups were evaluated using Student’s *t* test, whereas comparisons among 3 or more groups were assessed using one-way analysis of variance (ANOVA) followed by Tukey’s post hoc test. All data are presented as mean ± SD, and *P* < 0.05 was considered statistically significant.

## Results

### Direct cellular reprogramming of HDFs into osteoblasts through coexpression of Oct4 and Cbfβ

Oct4 and Cbfβ are hypothesized to play complementary roles in reprogramming somatic cells into osteoblasts, with Oct4 inducing a plastic state and Cbfβ promoting osteogenic differentiation. Based on these functions, we postulated that coexpression of Oct4 and Cbfβ could synergistically drive the direct reprogramming of HDFs into functional osteoblasts.

**Fig. 2.**
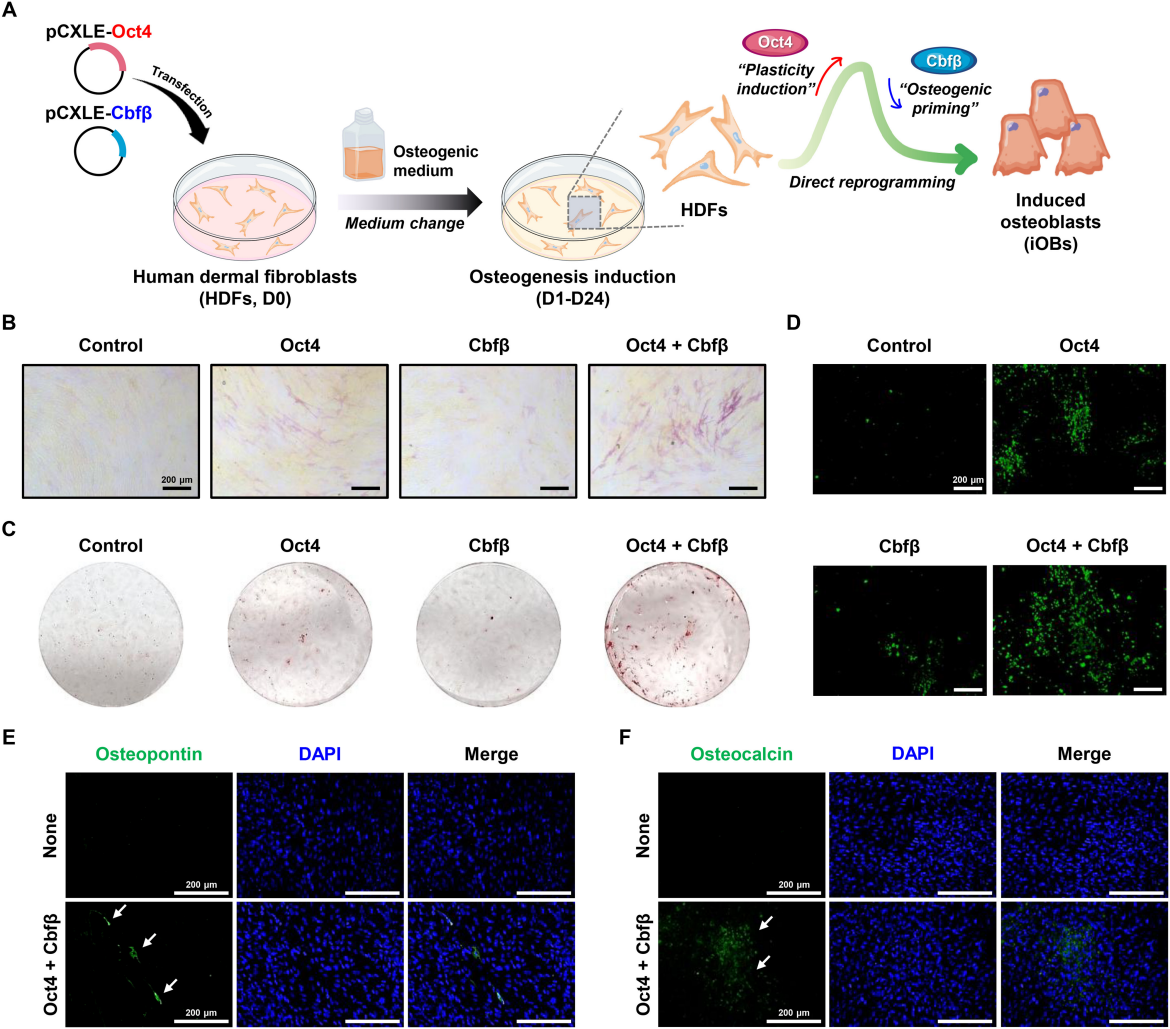
Direct reprogramming of HDFs into osteoblasts through ectopic overexpression of Oct4 and Cbfβ. (A) Schematic illustration of direct reprogramming process using the pCXLE episomal plasmid delivery system. The pCXLE-Oct4 and pCXLE-Cbfβ plasmids were delivered into HDFs via cationic polymer-based transfection, either individually or in combination. (B) Representative images of alkaline phosphatase (ALP) staining after 14 d of culture in osteogenic medium (OM). (C and D) Calcium deposition after 24 d of osteogenic induction, as detected by Alizarin Red S (ARS) and OsteoImage assays. (E and F) Immunofluorescence images showing expression of osteopontin (OPN) and osteocalcin (OCN), on day 24. Coexpression of Oct4 and Cbfβ induced robust expression of both osteogenic markers.

To test this hypothesis, we used the pCXLE episomal plasmid system, which minimized the risk of genomic integration [26]. pCXLE-Oct4 and pCXLE-Cbfβ, specifically designed to encode Oct4 and Cbfβ, were introduced into HDFs via cationic polymer-based transfection, either individually or in combination. After 24 d of transfection and culture under osteogenic conditions (Fig. 2A), we assessed the direct reprogramming of HDFs into osteoblasts by measuring ALP activity, an early osteogenic marker (Fig. 2B). Cbfβ alone elicited minimal ALP activity, whereas Oct4 alone produced a modest increase. In contrast, simultaneous overexpression of Oct4 and Cbfβ led to a significant elevation in ALP activity, indicating that coexpression of these 2 factors can directly reprogram HDFs into osteoblasts. We then evaluated osteoblast maturation of the resulting cells by examining mineral deposition using ARS staining (Fig. 2C) and OsteoImage mineralization assay (Fig. 2D). Consistent with the ALP staining results, the highest level of mineralization was observed in HDFs coexpressing Oct4 and Cbfβ, exceeding that of nontransduced controls or cells expressing either factor alone. Immunostaining analysis for OPN, an early-to-intermediate marker, and OCN, a specific marker of mature osteoblasts, demonstrated that coexpression of Oct4 and Cbfβ facilitated the generation of osteoblast-like cells positive for both key proteins (Fig. 2E and F). The robust expression of the late-stage marker OCN, concomitant with mineral deposition (Fig. 2C), confirms that the reprogrammed cells progressed beyond early progenitor stages to achieve functional maturation. Notably, only a subset of the total cell population acquired mature osteoblast characteristics, which is likely due to the limited transfection efficiency of polymer-mediated delivery of episomal plasmids and the consequent restricted expression of Oct4 and Cbfβ.

### Production and cytotoxicity assessment of recombinant Oct4/Cbfβ proteins fused with cell-permeable 30Kc19

**Fig. 3.**
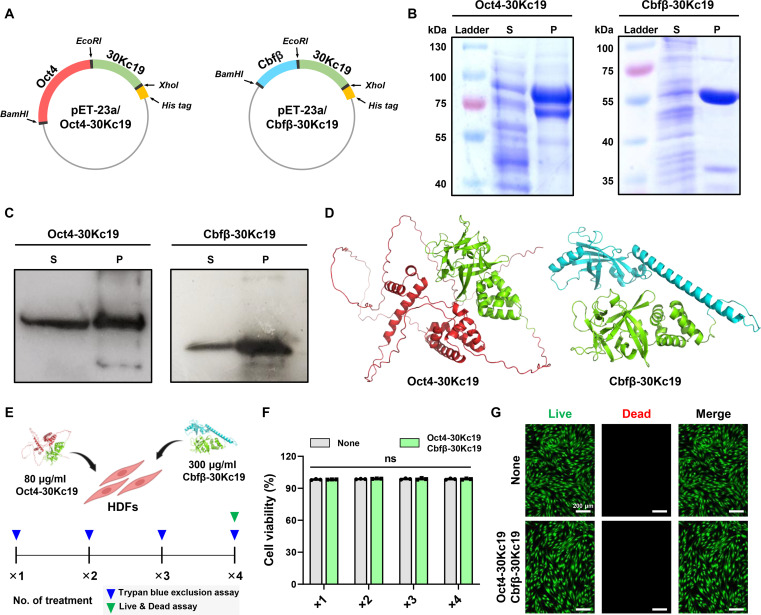
Expression, purification, and cytotoxicity assessment of recombinant reprogramming factors fused with 30Kc19. (A) Schematic representation of plasmid constructs for Oct4-30Kc19 and Cbfβ-30Kc19. The 30Kc19 protein was fused to the C terminus of each reprogramming factor proteins. (B and C) Coomassie blue staining and Western blot analysis of purified Oct4-30Kc19 and Cbfβ-30Kc19 proteins. S, soluble cell lysate; P, purified proteins. (D) Predicted 3D structures of Oct4-30Kc19 and Cbfβ-30Kc19 fusion proteins generated using AlphaFold2. Oct4 is shown in red, Cbfβ in cyan, and 30Kc19 in green. (E) Experimental timeline for cytotoxicity evaluation. HDFs were treated with Oct4-30Kc19 (80 μg/ml) and Cbfβ-30Kc19 (300 μg/ml) up to 4 times. Created with biorender.com. (F) Cytotoxicity analysis based on trypan blue exclusion assay after repeated protein treatment. Data are presented as means ± SD (*n* = 3). Statistical significance was determined by one-way ANOVA followed by Tukey’s post hoc test (ns, not significant). (G) Representative Live/Dead fluorescence images of HDFs following 4 consecutive protein treatments.

The large-scale production of recombinant transcription factors in soluble form using the *E. coli* system is often hindered by their physicochemical properties and inherent instability. To address this challenge, we constructed *E. coli* expression vectors for producing Oct4 and Cbfβ fused at their C terminus with the 30Kc19 protein, which was previously been shown to enhance soluble expression and stability (Fig. 3A) [13,23]. Following expression and purification, we analyzed the recombinant fusion proteins, Oct4-30Kc19 and Cbfβ-30Kc19, by Coomassie blue staining and Western blotting (Fig. 3B and C). Consistent with our previous findings [23], Cbfβ-30Kc19 was obtained in large quantities as a soluble form. In contrast, the intact form of Oct4 is typically expressed as insoluble inclusion bodies in *E. coli*, as described in several reports. However, fusion with 30Kc19 significantly improved the soluble expression of Oct4, consistent with our earlier observations [12]. Conjugation with 30Kc19 not only enhances the soluble expression of these transcription factors in the *E. coli* system but also improves their in vitro stability after purification, which is attributable to the protein-stabilizing properties of 30Kc19 [11,27].

To determine whether 30Kc19 conjugation altered proper protein folding, we predicted the 3D structures of Oct4-30Kc19 and Cbfβ-30Kc19 using an artificial intelligence system, AlphaFold2 [28]. The predicted structures suggested that the fusion with 30Kc19 did not significantly disrupt the native 3D conformation of Oct4 or Cbfβ compared with their intact forms (Fig. 3D and Movies S1 and S2). Although these predictive results cannot confirm that the transcription factors retained their full functionality for binding to their genomic targets, they support the notion that conjugation with 30Kc19 preserves the overall tertiary structure.

We next evaluated the cytotoxicity of recombinant protein treatment on HDFs to define an optimal treatment window. To overcome intracellular delivery barriers and maximize reprogramming efficiency, we utilized high protein concentrations previously established as the maximal nontoxic dose: 80 μg/ml for Oct4-30Kc19 and 300 μg/ml for Cbfβ-30Kc19 [13,23]. Cells were simultaneously exposed to 80 μg/ml of Oct4-30Kc19 and 300 μg/ml of Cbfβ-30Kc19, administered simultaneously for up to 4 consecutive treatments at 1-d intervals (Fig. 3E). Trypan blue exclusion assay revealed no significant cytotoxic effects under these conditions (Fig. 3F). Live/Dead staining further confirmed negligible cell death, even after repeated treatments at the tested concentrations (Fig. 3G). These results demonstrated that Oct4-30Kc19 and Cbfβ-30Kc19 can be administrated at relatively high doses with minimal cytotoxicity.

### Cell-penetrating properties and intracellular stability of 30Kc19-conjugated reprogramming factors

**Fig. 4.**
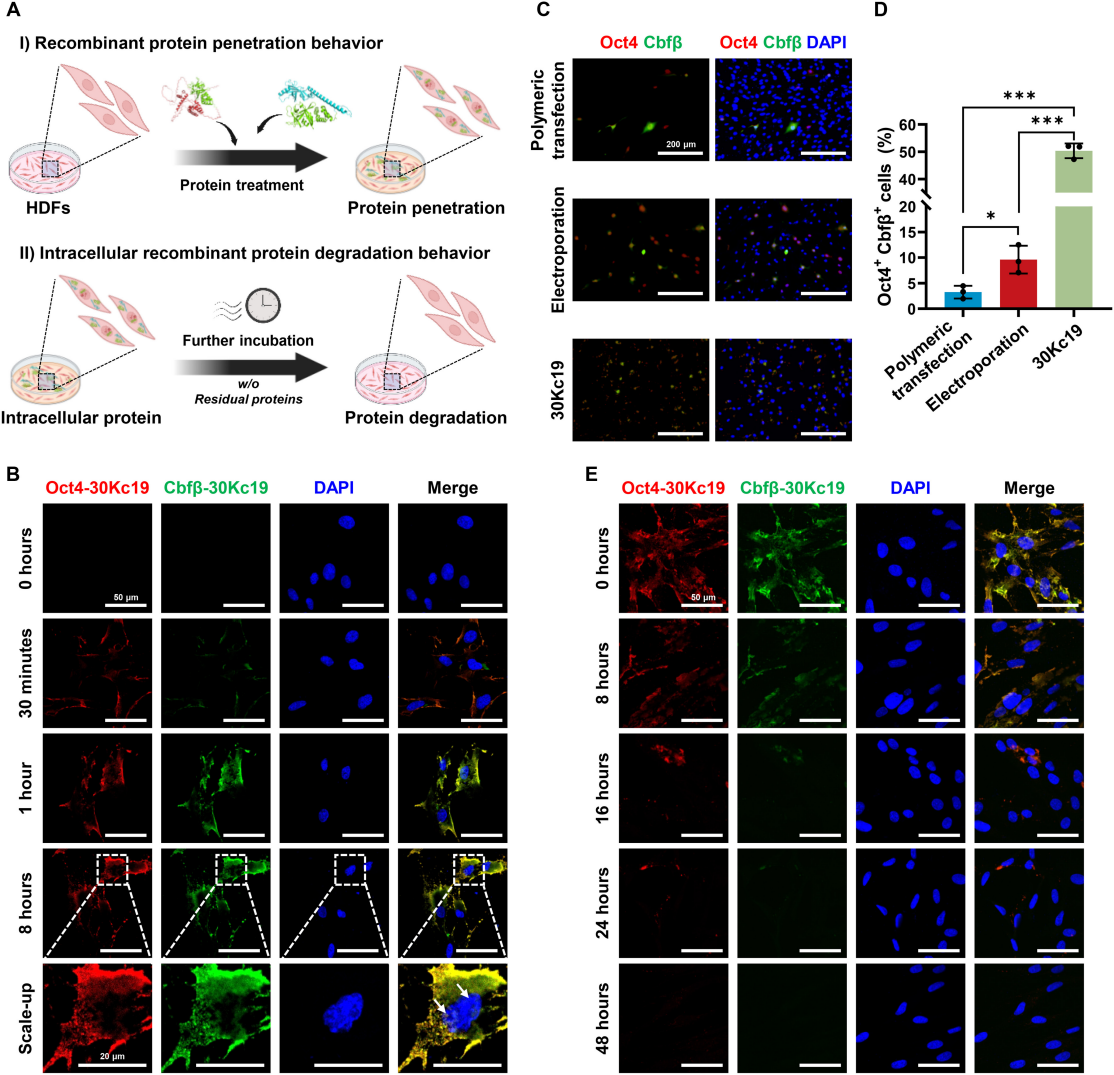
Efficient cellular uptake and intracellular stability of 30Kc19-fused reprogramming factor proteins. (A) Schematic illustration of the experimental design for assessing cellular uptake and intracellular stability of Oct4-30Kc19 and Cbfβ-30Kc19 proteins. Created with biorender.com. (B) Time-course immunofluorescence images depicting the progressive intracellular localization of Oct4-30Kc19 and Cbfβ-30Kc19 at 30 min, 1 h, and 8 h post-treatment. White arrows indicate nuclear localization of the recombinant proteins. (C and D) Comparison of delivery efficiency between 30Kc19-fused protein treatment and episomal plasmid transfection (via polymeric reagent and electroporation). A higher proportion of HDFs showed coexpression of Oct4 and Cbfβ following 30Kc19 protein-based delivery. Data are presented as means ± SD (*n* = 3). Statistical significance was determined by one-way ANOVA followed by Tukey’s post hoc test (**P < 0.05; ***P < 0.001). (E) Immunofluorescence images showing intracellular stability of Oct4-30Kc19 and Cbfβ-30Kc19 proteins up to 48 h after removal of residual proteins.

To investigate the intracellular behavior of these fusion proteins, we simultaneously treated HDFs with Oct4-30Kc19 and Cbfβ-30Kc19 and observed protein internalization via immunofluorescence staining (Fig. 4A). Thirty minutes post-treatment, both proteins were predominantly localized at or near the cell membrane (Fig. 4B). After 1 h, these proteins were broadly distributed throughout the cytoplasmic region, and after 8 h, a fraction was translocated to the nucleus while remaining detectable in the cytoplasm. These observations indicate that 30Kc19 effectively mediated cellular uptake and facilitated intracellular trafficking of Oct4 and Cbfβ.

For direct cellular reprogramming, the prescribed combination of reprogramming factors must be delivered simultaneously into the same cell. Unlike established iPSCs, which possess indefinite self-renewal capacity and can be derived from a small fraction of successfully transduced cells, directly reprogrammed cells have limited proliferative capacity. Therefore, efficient co-delivery of reprogramming factors into a large fraction of the starting cell population is essential. To compare delivery efficiency, we examined HDFs transfected with pCXLE-Oct4 and pCXLE-Cbfβ plasmids via polymeric transfection or electroporation. In those conditions, coexpression of Oct4 and Cbfβ was detected only in a limited percentage of the cell population (Fig. 4C and D). Although cationic polymer-based transfection is highly effective in certain cell lines, its efficiency varies significantly between cell types. As shown in Fig. 2, direct reprogramming of HDFs into functional osteoblasts was feasible with DNA plasmid transfection, but at a relatively low efficiency, likely due to limited transfection efficiency. In contrast, when HDFs were treated with Oct4-30Kc19 and Cbfβ-30Kc19, we detected co-delivery of both proteins in the majority of cells (Fig. S1). These results demonstrate that 30Kc19-mediated protein delivery achieved considerably higher efficiency of simultaneous factor introduction compared with plasmid-based methods.

Next, we examined the intracellular stability of 30Kc19-conjugated reprogramming factors (Fig. 4A). Previous cell-free studies have shown that 30Kc19 fusions remain soluble and resistant to aggregation and proteolysis at 37 °C [13]. To investigate whether these properties are maintained in a cellular context, HDFs were treated with Oct4-30Kc19 and Cbfβ-30Kc19 for 8 h, followed by medium replacement to remove residual extracellular proteins. Immunostaining revealed that although the intracellular protein levels gradually decreased, both fusion proteins remained detectable for up to 24 h after treatment without additional supplementation (Fig. 4E). These data indicate that 30Kc19 not only enables efficient cellular entry of reprogramming factors but also supports their day-scale intracellular persistence and nuclear localization. Importantly, this physical stability is closely associated with functional efficacy. Previous studies utilizing luciferase reporter assays have reported that 30Kc19-fused reprogramming factors retain robust transcriptional activity over extended periods [12,13]. This suggests that 30Kc19-mediated intracellular retention provides a sufficient time window for the delivered factors to bind target genomic sites and initiate the reprogramming cascade before degradation. Taken together, these properties align with the functional requirements for direct lineage conversion, highlighting 30Kc19 as an effective fusion partner for protein-based reprogramming strategies.

### Generation of functional osteoblasts by treatment of HDFs with Oct4-30Kc19 and Cbfβ-30Kc19 proteins

**Fig. 5.**
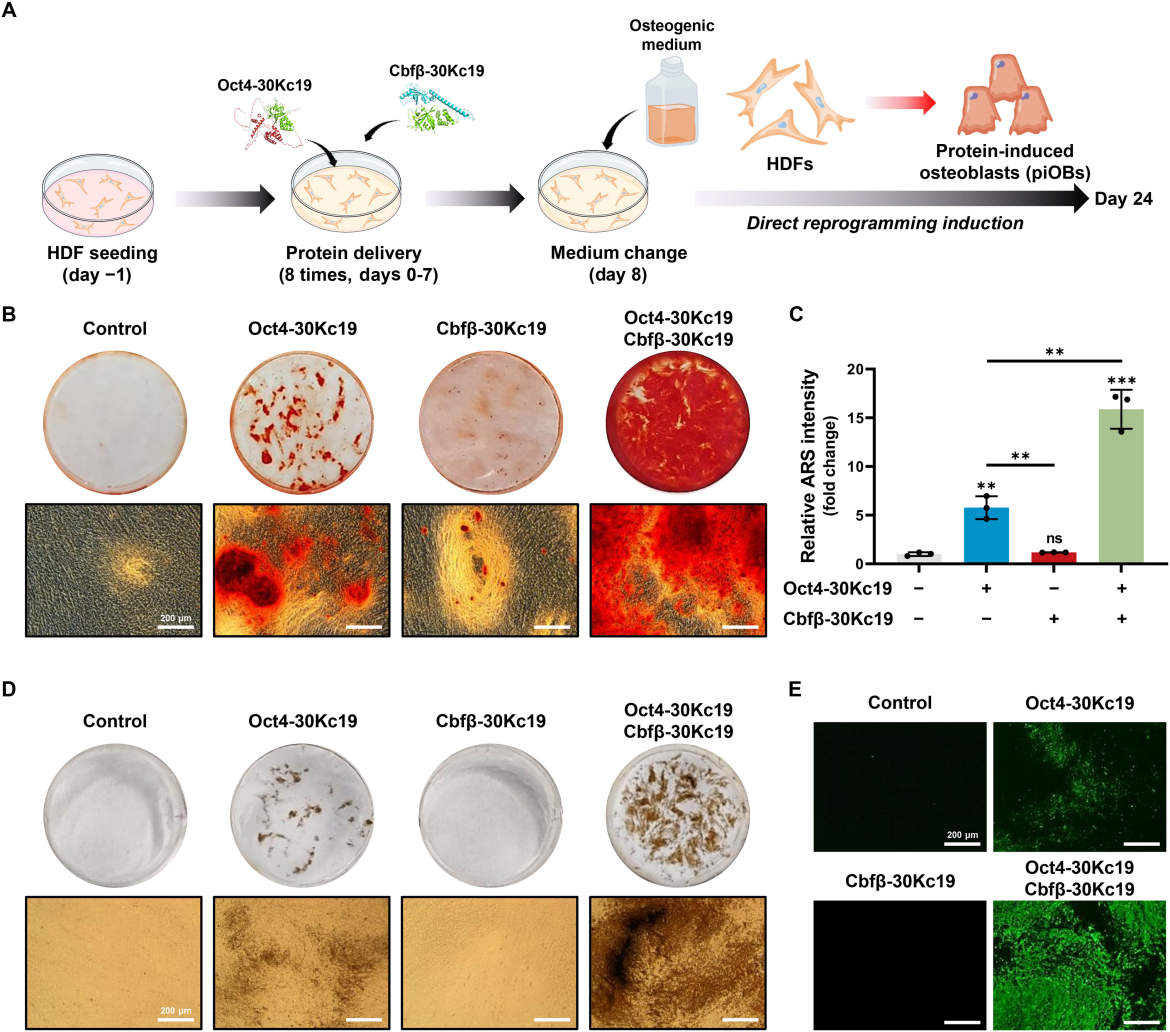
Direct reprogramming of HDFs into osteoblasts using cell-permeable protein-based platform. (A) Schematic illustration of the reprogramming strategy using 30Kc19-fused recombinant proteins. HDFs were treated with a combination of Oct4-30Kc19 and Cbfβ-30Kc19 recombinant proteins 8 times over 8 d, followed by culture in OM. (B and C) ARS staining and subsequent quantification on day 24, showing robust calcium deposition in the group treated with both proteins. Data are presented as means ± SD (*n* = 3). Statistical significance was determined by one-way ANOVA followed by Tukey’s post hoc test. Unless otherwise indicated, comparisons were made to the nontreated control group (**P < 0.01; ***P < 0.001; ns, not significant). (D and E) Von Kossa staining and OsteoImage mineralization assay images, confirming mineralized matrix formation. Treatment with both Oct4-30Kc19 and Cbfβ-30Kc19 resulted in significantly enhanced mineralization compared with single-factor or untreated controls.

Building on the observed intracellular delivery and stability of these fusion proteins, we performed direct reprogramming of HDFs by administering Oct4-30Kc19 and Cbfβ-30Kc19 8 consecutive times, followed by culture in OM (Fig. 5A). By day 24 of osteogenic reprogramming, ARS staining revealed the robust formation of mineralized matrices in cells treated with both proteins, indicating successful generation of functional osteoblasts. Consistent with the results from the episomal DNA approach, treatment with either Oct4-30Kc19 or Cbfβ-30Kc19 alone produced minimal mineralization, whereas cotreatment significantly increased calcium deposition (Fig. 5B). Quantitative analysis showed that cotreatment resulted in approximately 15.87-fold higher calcium deposition compared with nontreated control and 2.74-fold higher than Oct4-30Kc19 treatment alone (Fig. 5C), demonstrating the synergistic osteogenic activity of Oct4 and Cbfβ. Another noteworthy point is that calcium deposition was significantly greater in the 30Kc19 fusion protein-based approach compared with the episomal DNA delivery system (Figs. 2C and 5B), indicating superior efficiency of 30Kc19-mediated co-delivery in direct osteogenic reprogramming.

Further evidence of mineralized matrix formation in the converted cells was obtained via von Kossa staining and OsteoImage mineralization assay (Fig. 5D and E). Quantitative assessments of these assays further demonstrated the superior mineralization efficiency of the cotreatment strategy (Fig. S2A and B). Both analyses confirmed negligible mineralization upon treatment with Cbfβ-30Kc19 alone, comparable to nontreated control HDFs, while Oct4-30Kc19 alone produced only modest mineral deposits. These findings suggest that Oct4 facilitates the induction of an epigenetically permissive, “plastic” state, but requires Cbfβ to achieve full osteogenic reprogramming. Collectively, these results clearly demonstrate that Oct4-mediated partial reprogramming must be complemented by Cbfβ to drive complete conversion to fully functional osteoblasts.

### Transcriptomic analysis of directly converted osteoblasts

**Fig. 6.**
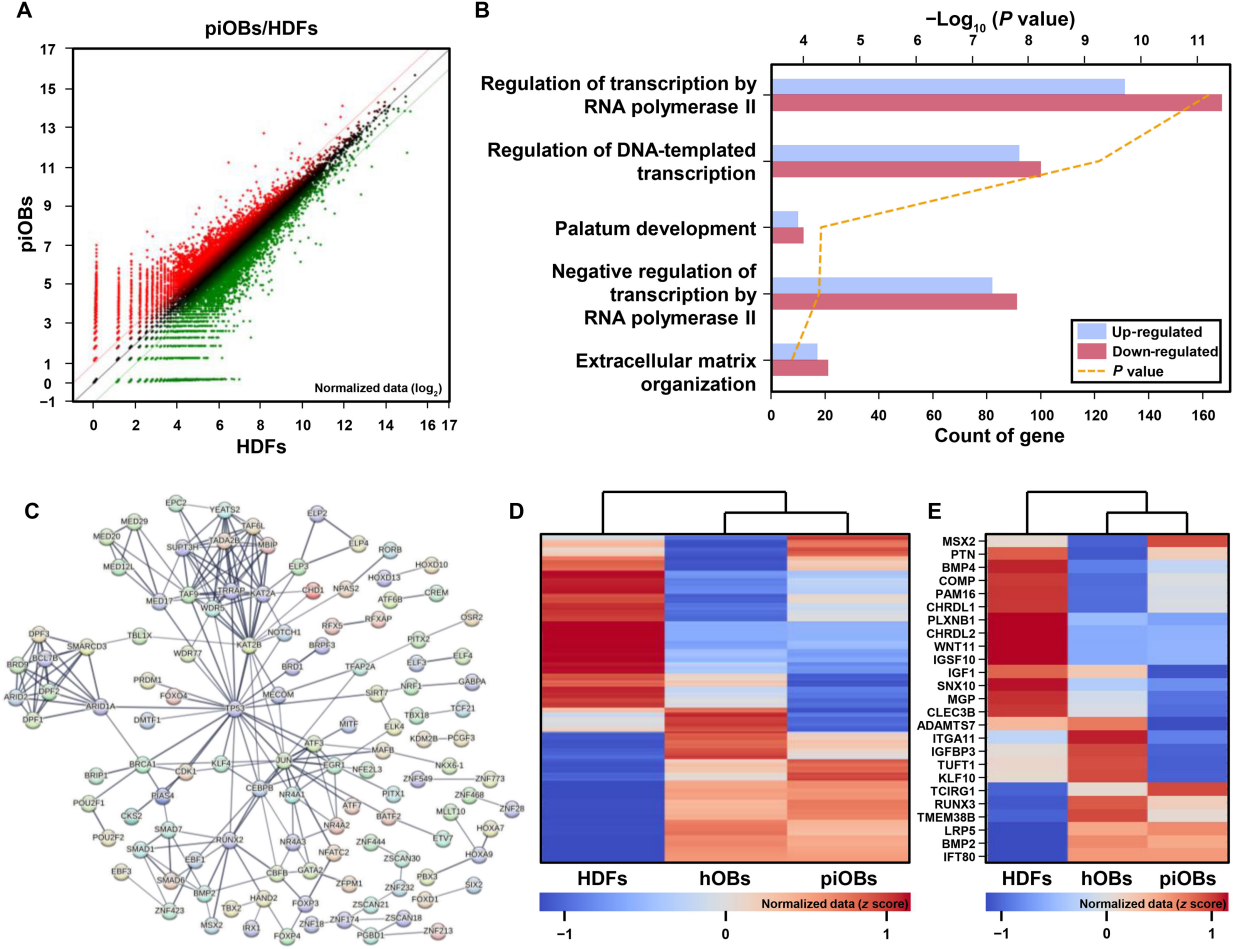
Transcriptomic remodeling toward an osteoblast lineage by Oct4-30Kc19 and Cbfβ-30Kc19 fusion proteins. (A) Scatterplot showing differentially expressed genes (DEGs) between nontreated HDFs and protein-induced osteoblasts (piOBs) treated with Oct4-30Kc19 and Cbfβ-30Kc19 proteins (fold change ≥ 2). (B) Gene Ontology (GO) enrichment analysis of DEGs, highlighting overrepresentation of transcription-related biological processes. (C) Protein interaction network of enriched GO terms related to transcription regulation by RNA polymerase II. (D) Hierarchical clustering analysis of genes differentially expressed in both piOBs and primary human osteoblasts (hOBs), relative to HDFs. (E) Heatmap of DEGs associated with the ossification GO term, showing the expression of key osteogenic markers.

To investigate global gene expression changes associated with protein-induced reprogramming, we performed QuantSeq 3′ mRNA sequencing of nontreated HDFs and piOBs generated by treatment with Oct4-30Kc19 and Cbfβ-30Kc19. All samples were collected on day 24 of osteogenic culture. Among the 27,758 identified transcripts, scatterplot analysis revealed 3,171 DEGs in piOBs compared with nontreated HDFs (fold change ≥ 2), of which 1,516 were up-regulated and 1,655 were down-regulated (Fig. 6A). GO enrichment analysis of these DEGs revealed significant overrepresentation of GO terms associated with transcriptional regulation (Fig. 6B). In particular, categories related to RNA polymerase II-mediated and DNA-templated transcriptional processes were enriched (Fig. 6C), suggesting broad reorganization of transcriptional activity during reprogramming. These findings are consistent with the known functions of Oct4 and Cbfβ. Oct4 is a pioneer transcription factor that induces chromatin remodeling and enhances transcriptional plasticity, while Cbfβ acts as a coactivator of Runx2, a key regulator of osteoblast-specific gene expression [29,30]. This enrichment likely reflects changes in transcription factor activity and chromatin accessibility, which collectively facilitate the activation of osteogenic gene expression programs.

To determine whether this transcriptional reprogramming resulted in the acquisition of an osteoblast-like identity, we utilized primary hOBs as an internal positive control. This approach allowed for a direct transcriptomic comparison while minimizing batch effects associated with external datasets. Hierarchical clustering of 1,584 genes that were differentially expressed in both piOBs and hOBs relative to HDFs revealed that piOBs clustered more closely with hOBs than with HDFs (Fig. 6D). Although the piOB sample was asynchronous, reflecting heterogeneity in reprogramming state, the bulk transcriptome clearly diverged from HDFs and shifted toward that of hOBs. Next, we examined the DEGs associated with ossification-related GO terms (Fig. 6E). Among these genes, bone morphogenetic protein 2 (BMP2), which is vital for bone and cartilage development and strongly promotes osteoblast differentiation in various types of stem cells, was up-regulated in piOBs [31,32]. TMEM38B, also known as trimeric intracellular cation channel type B (TRIC-B), a monovalent cation channel primarily localized to the membrane of the endoplasmic reticulum and required for calcium homeostasis and osteoblast mineralization [33], was also up-regulated. Mutations in *TMEM38B* impair calcium handling and compromise bone formation, highlighting the relevance of its induction in piOBs [34,35]. Despite expected heterogeneity, clustering of these ossification-related genes demonstrated greater similarity between piOBs and hOBs, supporting acquisition of an osteoblast-like transcriptional program. Although the limited sample size precludes rigorous statistical analysis at the single-gene level, the global profile provides compelling descriptive evidence of lineage conversion. Collectively, these findings indicate that treatment with Oct4-30Kc19 and Cbfβ-30Kc19 induces extensive transcriptional reprogramming of HDFs, driving their conversion toward an osteoblast-like identity.

### In vivo bone formation by implanted piOBs

To evaluate the bone-forming capacity of piOBs in vivo, we established a critical-sized calvarial defect model in mice and implanted piOBs into the defect. As a transplantation scaffold, we used a gelatin cryogel with interconnected macroporosity, known for its high biocompatibility, mechanical stability, and ability to support in-tissue growth (Fig. S3A) [36]. SEM images revealed a porous structure of the gelatin cryogel with approximately 45% interconnectivity (Fig. S3B and C). This porous network promotes nutrient and oxygen exchange, thereby enhancing key cellular activities, such as proliferation, migration, and angiogenesis [37]. In addition, the gelatin cryogel exhibited a crosslinking degree of approximately 45%, ensuring structural integrity while permitting cell infiltration through its swelling capacity (Fig. S3D and E). When seeded with HDFs, metabolic activity continuously increased over the 7-d incubation period, and Live/Dead staining demonstrated negligible cytotoxicity, confirming that the cryogel provided a cell-supportive microenvironment (Fig. S4A and B). These results emphasize the suitability of gelatin cryogels as scaffolds for piOB transplantation.

**Fig. 7.**
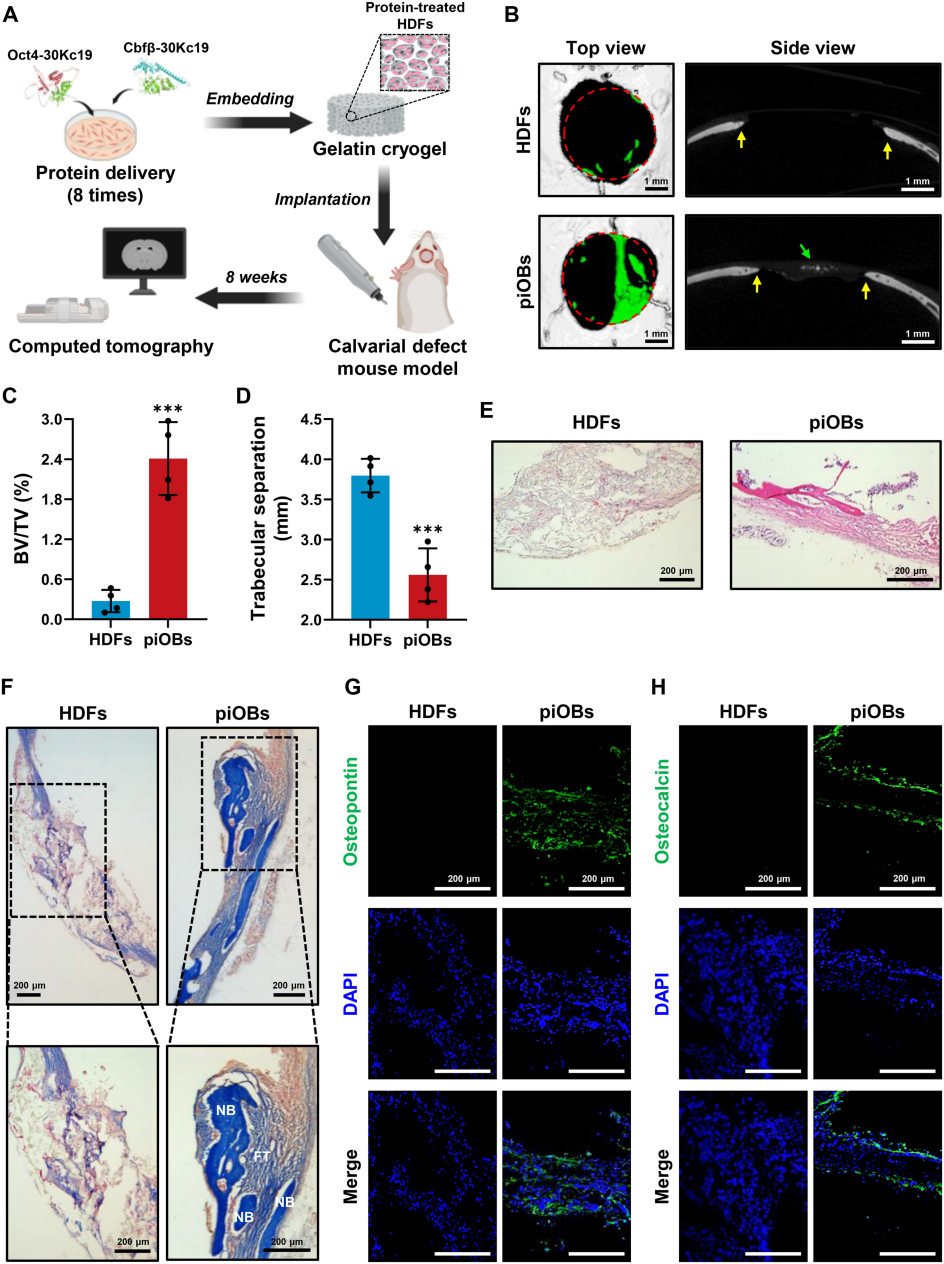
Efficient bone defect regeneration using a cell-permeable protein-based direct reprogramming platform. (A) Schematic illustration of the in vivo bone regeneration experiment. HDFs were pretreated 8 times with Oct4-30Kc19 and Cbfβ-30Kc19 proteins, seeded onto gelatin cryogels, and transplanted into 4-mm-sized calvarial defects in mice. Created with biorender.com. (B) Representative micro-CT 3D images showing bone regeneration 8 weeks post-transplantation. Green areas and arrows indicate newly regenerated bones, while yellow arrows denote bone defect regions. (C and D) Quantification of bone volume fraction (BV/TV) and trabecular separation in the regenerated bone tissue. Data are presented as means ± SD (*n* = 4). Statistical significance was determined by Student’s *t* test (***P < 0.001). (E) Hematoxylin and eosin (H&E) staining showing histological differences between defects implanted with untreated HDFs and those implanted with piOBs. (F) Masson’s trichrome (MTC) staining revealing collagen-rich new bone formation in the piOB-treated group. FT, fibrous tissues; NB, new bones. (G and H) Immunofluorescent staining for OPN and OCN, confirming the presence of mature osteoblast-derived matrix in piOB-transplanted defects.

For implantation, HDFs were pretreated with Oct4-30Kc19 and Cbfβ-30Kc19 for 8 consecutive cycles, seeded onto the gelatin cryogel, and transplanted into the defect site created on the calvaria of mice (Fig. 7A). Before evaluating the cellular contribution, we verified that the gelatin cryogel-only group exhibited negligible bone formation (Fig. S5), indicating that the scaffold itself lacks intrinsic osteo-inductive capacity. Subsequently, micro-CT 3D imaging (Fig. 7B) and quantitative analysis of BV/TV and trabecular separation demonstrated a significant increase in bone formation and a corresponding reduction in defect size in the piOB-implanted group compared with the control group seeded with nontreated HDFs (Fig. 7C and D). Histological analysis further substantiated in vivo bone regeneration following the transplantation of piOBs. H&E staining of the sagittal section revealed dense bone tissue bridging the entire defect region in piOB grafts, whereas loose connective tissue was predominant in controls (Fig. 7E). MTC staining indicated abundant, blue-stained collagen deposition in the piOB-implanted defects, providing additional evidence for new bone formation (Fig. 7F). Moreover, immunohistochemical staining showed prominent expressions of OPN and OCN, 2 well-established osteoblast markers, in the piOB-transplanted group, but negligible staining in the control group (Fig. 7G and H). Collectively, these in vivo findings demonstrate that the treatment of HDFs with Oct4-30Kc19 and Cbfβ-30Kc19 induces their direct conversion into functional osteoblasts capable of regenerating bone tissue in vivo in a manner comparable to endogenous osteoblasts.

## Discussion

The present study introduces a novel protein-based strategy for direct osteoblastic conversion, achieved by fusing the reprogramming factors Oct4 and Cbfβ with the cell-permeable and protein-stabilizing 30Kc19 protein. This approach addresses key limitations of current reprogramming methods, providing a potentially safer and more efficient alternative for osteoblast generation, with implications for bone regenerative medicine.

In the *E. coli* expression system, both factors were expressed in fusion with 30Kc19, which enhanced their solubility and facilitated efficient cellular uptake. Compared with plasmid DNA delivery through cationic polymer-based transfection or electroporation, Oct4-30Kc19 and Cbfβ-30Kc19 proteins achieved internalization into a greater proportion of HDFs (Fig. 4), resulting in enhanced osteogenic conversion, as evidenced by ARS staining (Figs. 2C and 5B). This superior efficiency is attributed not only to enhanced intracellular delivery but also to the stability conferred by the fusion with 30Kc19, facilitating prolonged intracellular retention, including nuclear localization (Fig. 4E). Previous work has shown that the conjugation of 30Kc19 to Yamanaka factors significantly improved their solubility in *E. coli* expression systems as well as their stability in both in vitro and intracellular environments; the same principle applies here to Oct4 and Cbfβ [12]. Furthermore, previous investigations utilizing the 30Kc19 α-domain, the functional moiety responsible for cellular uptake, confirmed that the carrier protein alone does not induce osteogenic differentiation or bone regeneration in both in vitro and in vivo models, supporting its role as an inert delivery vehicle lacking intrinsic osteogenic activity [38]. Although protein-based reprogramming provides a safer alternative without genomic integration, it generally requires repeated administration of high protein concentrations because of the inherent instability of proteins. This limitation presents a particular challenge for generating numerous target cells through protein-based reprogramming. The enhanced solubility and stability of Oct4-30Kc19 and Cbfβ-30Kc19 proteins suggest that efficient reprogramming can be achieved with reduced protein doses and fewer administrations, thereby increasing feasibility for both in vitro protocols and potential in vivo applications.

Another notable advancement of this study is the successful conversion into osteoblasts using only a combination of 2 reprogramming factors, Oct4 and Cbfβ, in contrast to a previous report that required 4 factors (Oct4, L-Myc, Runx2, and Osterix) [20]. Considering the complex physicochemical properties of proteins, minimizing the number of required proteins simplifies manufacturing protocols and may improve overall reprogramming efficiency. Protein-mediated iPSC reprogramming, particularly in nascent stages, exhibited extremely low efficiency compared to genetic methods, partly due to the extensive epigenetic remodeling and prolonged factor expression required to achieve full pluripotency [39,40]. In contrast, indirect evidence suggests that direct lineage conversion demands only transient or lower expression of reprogramming factors, rendering protein-based strategies particularly advantageous. Importantly, our approach achieved osteoblast-specific reprogramming without relying on exogenous growth factors or complex small-molecule cocktails, which may inadvertently activate multiple signaling pathways and cause off-target differentiation. In a recent study, the direct lineage conversion of human vascular endothelial cells into osteoblasts was achieved by coadministering Oct4-30Kc19 fusion protein, as used in the present study, and BMP4, a growth factor associated with the activation of the SMAD signaling pathway [41]. Although BMP4 has been used to induce osteogenesis in various cell types, its pleiotropic effects on cellular signaling pathways and multilineage differentiation raise concerns regarding nonspecific differentiation [42,43]. By relying on the specific combination of Oct4 and Cbfβ, our approach minimizes the potential for nonspecific lineage commitment.

Mechanistically, we propose that the direct reprogramming observed in this study is driven by a cooperative process involving Oct4-mediated plasticity induction coupled with Cbfβ-driven osteogenic priming. Oct4 is widely recognized as a pioneer transcription factor that initiates reprogramming by inducing a “plastic state”, characterized by the down-regulation of somatic identity and increased responsiveness to environmental cues [19,44]. Consistent with this, our in vitro results showed that treatment with Oct4-30Kc19 alone induced modest calcium deposition (Fig. 5), suggesting that Oct4 primes the fibroblasts into a permissive state. To achieve complete lineage conversion, the synergistic action of Cbfβ was essential to direct this plastic potential toward a fully functional osteogenic fate [21]. Chemical reprogramming represents another promising paradigm, with several studies reporting direct osteogenic conversion of fibroblasts using defined cocktails of small molecules [45,46]. This approach presents distinct advantages, including simplified manufacturing and standardization, reduced immunogenic potential, and the obviation of genomic integration. However, its efficacy is critically dependent on precise optimization of the composition, concentration, and temporal application of each constituent small molecule throughout the differentiation trajectory. Suboptimal dosing or imprecise combinatorial regimens can result in significant cytotoxicity or impair the functional maturation of the resultant induced osteoblasts. Furthermore, the potential for promiscuous activation of diverse intracellular signaling cascades by such cocktails carries the risk of inadvertently stimulating off-target pathways, thereby perturbing cellular homeostasis and potentially leading to nonspecific lineage commitment or even tumorigenesis.

Our research further demonstrates the versatility of this platform not only for in vitro direct reprogramming but also through a novel approach combining partial pre-reprogramming with subsequent in vivo maturation. In contrast to conventional methods that rely on fully reprogrammed cells before transplantation, our approach harnesses endogenous biochemical and biophysical cues within the host microenvironment to orchestrate the terminal maturation of partially primed cells [47,48]. Such in vivo maturation phase may improve cellular integration, maturation, and long-term functional stability by exploiting native physiological signals. Notably, the substantial bone regeneration observed over 8 weeks in the calvarial defect model, without additional protein supply, demonstrates that the induced osteogenic identity is stably sustained in the physiological environment. At the molecular level, Oct4-30Kc19 and Cbfβ-30Kc19 act synergistically to remodel transcriptional networks, thereby converting somatic cells toward an osteogenic fate. This effect was further enhanced through the use of an interconnected macroporous gelatin cryogel scaffold, which provided structural support, facilitated nutrient and oxygen diffusion, and promoted the stable engraftment of transplanted cells [49]. By combining partial pre-reprogramming with a tailored biomaterial scaffold, our platform achieved superior regenerative outcomes, offering a synergistic advantage for clinical translation. Given that bone regeneration relies on a complex physiological milieu, the dynamic interplay between transplanted cells and host cell populations, including endogenous osteoblasts and osteoclasts, act as a critical determinant of the overall regenerative efficacy. Although detailed analysis of these interactions was beyond the scope of this study, elucidating the specific crosstalk between transplanted piOBs and the host microenvironment will be essential for fully comprehending the mechanisms of tissue regeneration and guiding further optimization.

In terms of future perspectives, although in vivo osteogenic reprogramming was not directly confirmed in the present study, the superior stability and cell permeability of Oct4-30Kc19 and Cbfβ-30Kc19 proteins support their potential utility for safe and efficient direct lineage conversion in vivo as well as in vitro. Additionally, while substantial bone regeneration was observed in our in vivo model, future investigations utilizing larger animal cohorts would be beneficial to further validate the statistical robustness and generalizability of these findings. Furthermore, the conjugation of these proteins with tags capable of targeting bone tissue, such as hydroxyapatite-binding peptides [50], could further enhance bone-specific localization and in vivo conversion efficiency, thereby advancing their clinical applicability.

In conclusion, we established a protein-based direct reprogramming platform that converts HDFs into functional osteoblasts through the delivery of only 2 reprogramming factors, Oct4 and Cbfβ, each fused to the *B. mori*-derived 30Kc19 protein. This method achieved superior intracellular delivery efficiency and stability with minimal cytotoxicity. Transcriptomic analysis revealed that the resulting piOBs share key transcriptomic features with primary hOBs, confirming successful osteogenic reprogramming. Moreover, in vivo transplantation of piOBs into a critical-sized calvarial defect in mice led to substantial bone regeneration, highlighting the therapeutic potential of our approach. By eliminating the need for viral vectors or exogenous genetic materials, and by requiring only 2 factors, our platform avoids safety concerns associated with conventional reprogramming methods. Collectively, our findings provide new possibilities for safer and more precise direct reprogramming strategies in regenerative medicine, particularly for the therapeutic applications in bone repair and regeneration.

## Data Availability

Data will be made available on request. The RNA-sequencing data have been deposited in the NCBI Gene Expression Omnibus (GEO) under accession number GSE292005.
