# Supplementary material for: Transgene-Free Direct Osteogenic Reprogramming Using Cell-Permeable Octamer-Binding Transcription Factor 4/Core-Binding Factor β Fusion Proteins
Source: Biomater Res. 2026 Feb 3;30:0320. doi: 10.34133/bmr.0320 (PMC12864657; doi:10.34133/bmr.0320)
Supplement: Supplementary 1 — Figs. S1 to S5 Movies S1 and S2 [file bmr.0320.f1.zip › Supplementary Materials (Clean Version).docx]

**Transgene-free direct osteogenic reprogramming using cell-permeable octamer-binding transcription factor 4/core-binding factor β fusion proteins**

Manho Kim^1^, Jaeyoung Lee^1^, Wijin Kim^1^, Songrae Kim^1,2^, Jongmin Park^3,4^, Ju Hyun Park^1,4*^

^1^ Department of Biomedical Science, Kangwon National University, Republic of Korea.

^2^ Metropolitan Seoul Center, Korea Basic Science Institute (KBSI), Republic of Korea.

^3^ Department of Chemistry, Kangwon National University, Republic of Korea.

^4^ Institute of Molecular Science and Fusion Technology, Kangwon National University, Republic of Korea.

*Address correspondence to: juhyunpark@kangwon.ac.kr


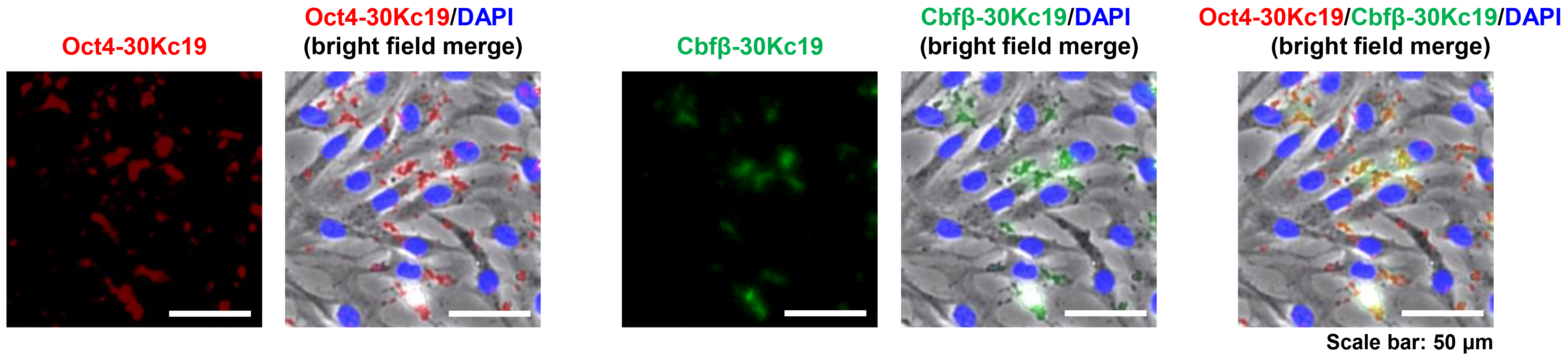


**Fig. S1.** Representative immunostaining image confirming intracellular co-localization of delivered Oct4-30Kc19 (red) and Cbfβ-30Kc19 (green) in human dermal fibroblasts (HDFs).


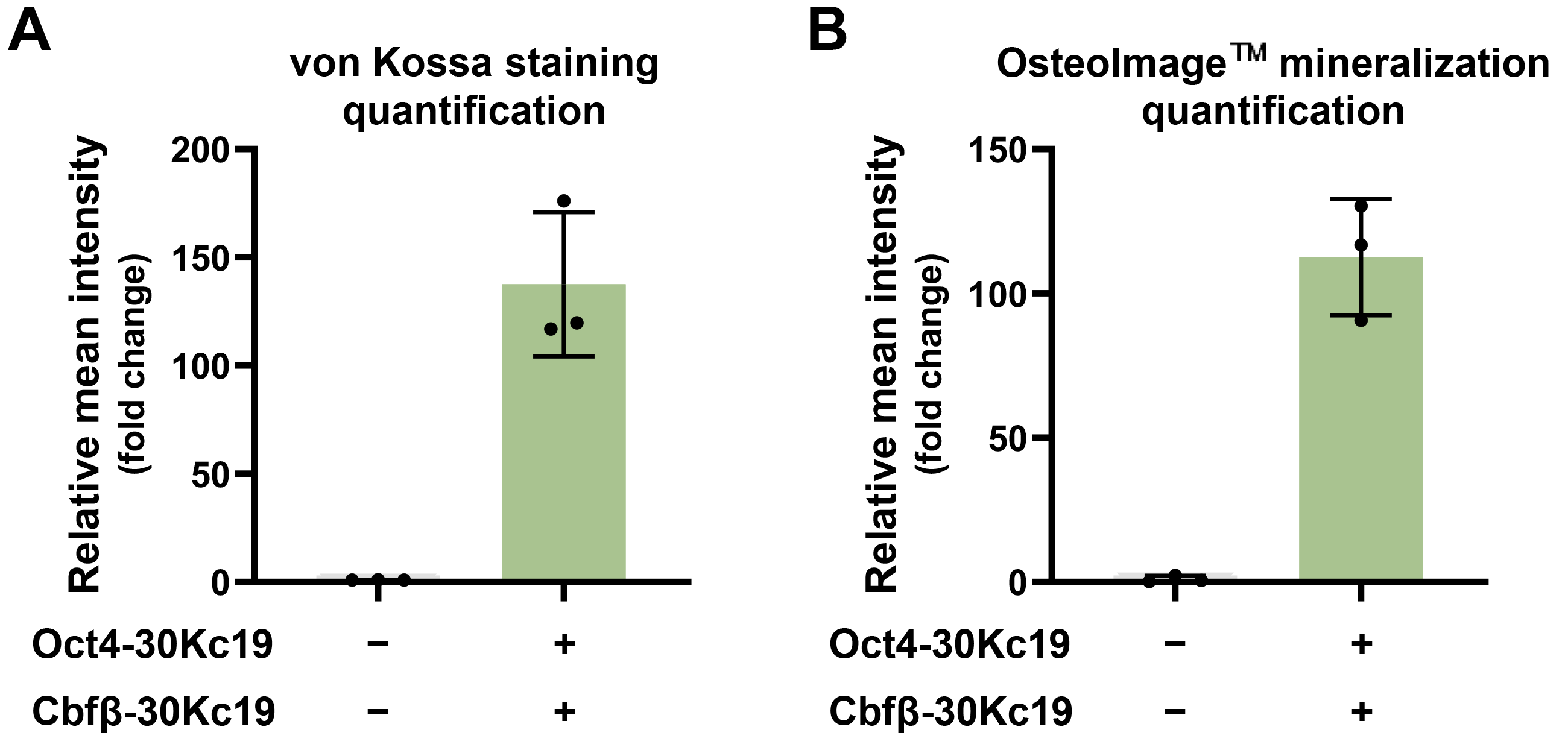


**Fig. S2.** Quantitative assessment of mineralized matrix deposition. (A) Relative quantification of calcium deposits determined by von Kossa staining. (B) Quantitative evaluation of mineralization efficiency using the OsteoImage™ mineralization assay. Data are presented as means ± SD (*n* = 3).


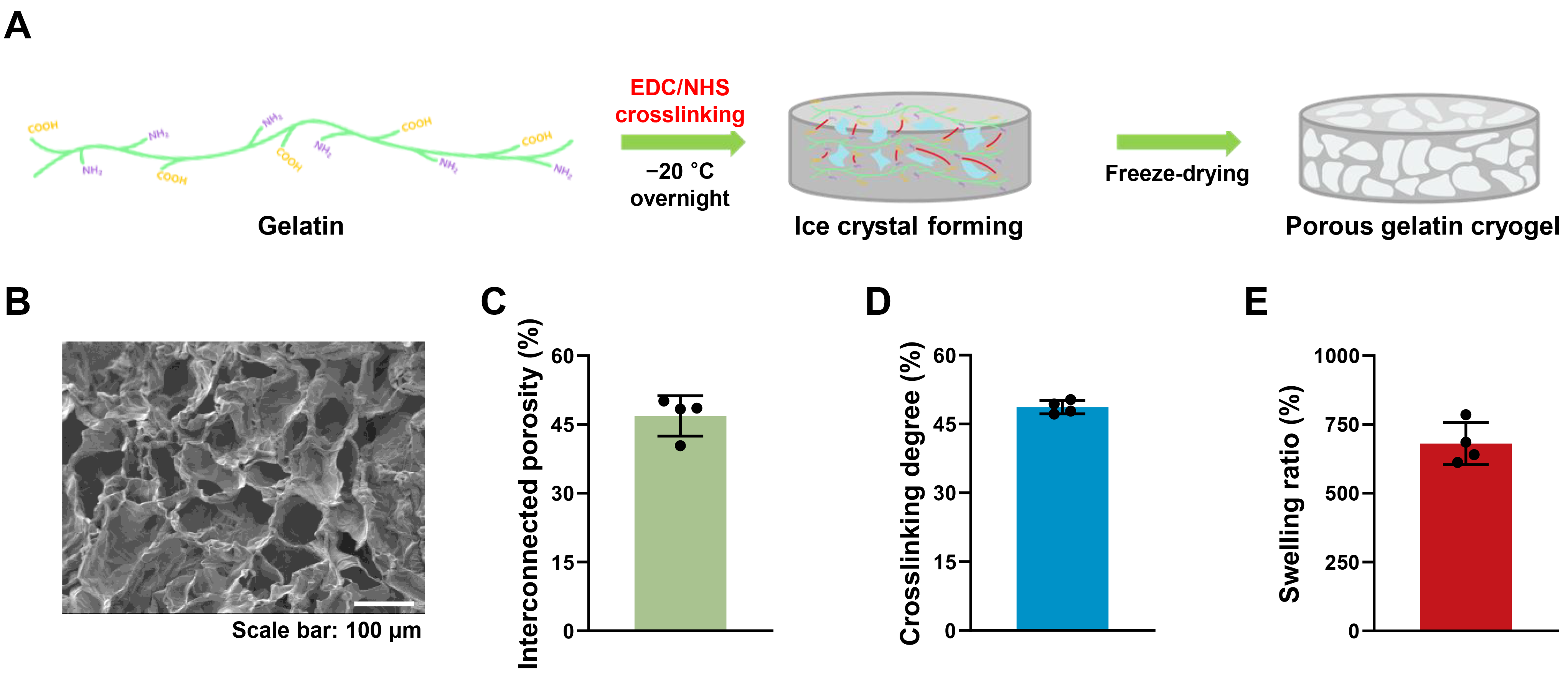


**Fig. S3.** Fabrication and characterization of gelatin cryogel. (A) Schematic illustration of the gelatin cryogel fabrication process. (B) Scanning electron microscopy (SEM) image showing the porous structure of the gelatin cryogel. (C) Interconnected porosity evaluated using the water wicking method. (D) Crosslinking degree assessed by ninhydrin assay. (E) Swelling ratio calculated based on the freeze-dried weight and swollen weight of the cryogel. All numerical data are presented as means ± SD (*n* = 4).


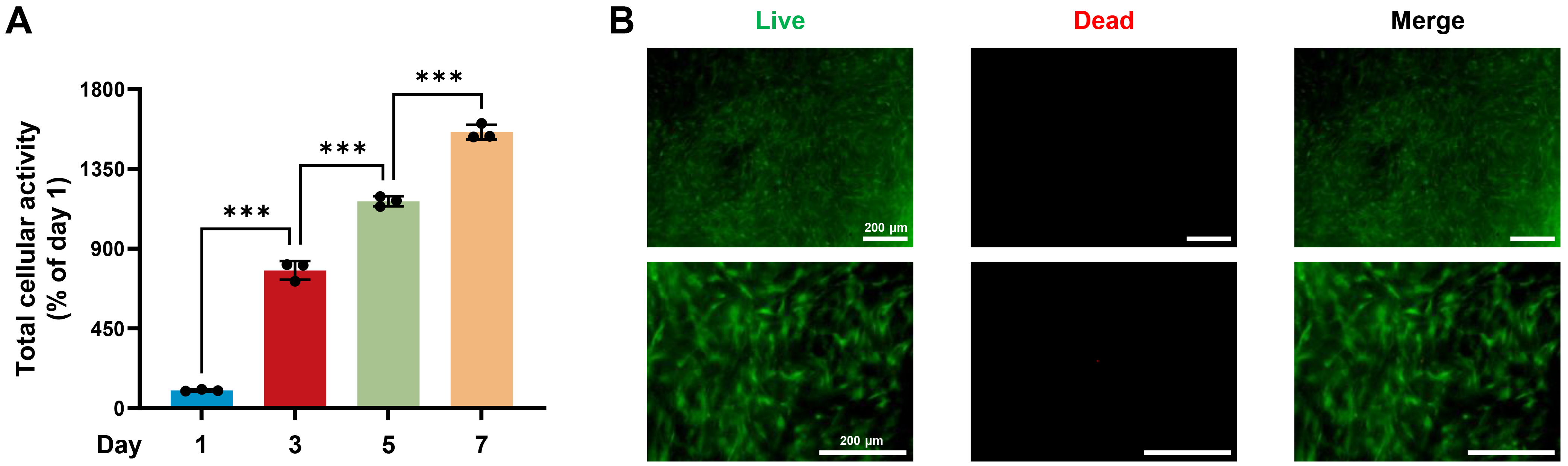


**Fig. S4.** Cytotoxicity evaluation of gelatin cryogel on human dermal fibroblasts (HDFs). (A) Cell proliferation of HDFs assessed by water-soluble tetrazolium salt-8 (WST-8) assay over a 7-day culture period. Data are presented as means ± SD (*n* = 3). Statistical significance was determined by one-way ANOVA followed by Tukey's post hoc test (****p* < 0.005). (B) Live/Dead fluorescence images of HDFs cultured in gelatin cryogel for 7 days, showing minimal cytotoxicity.


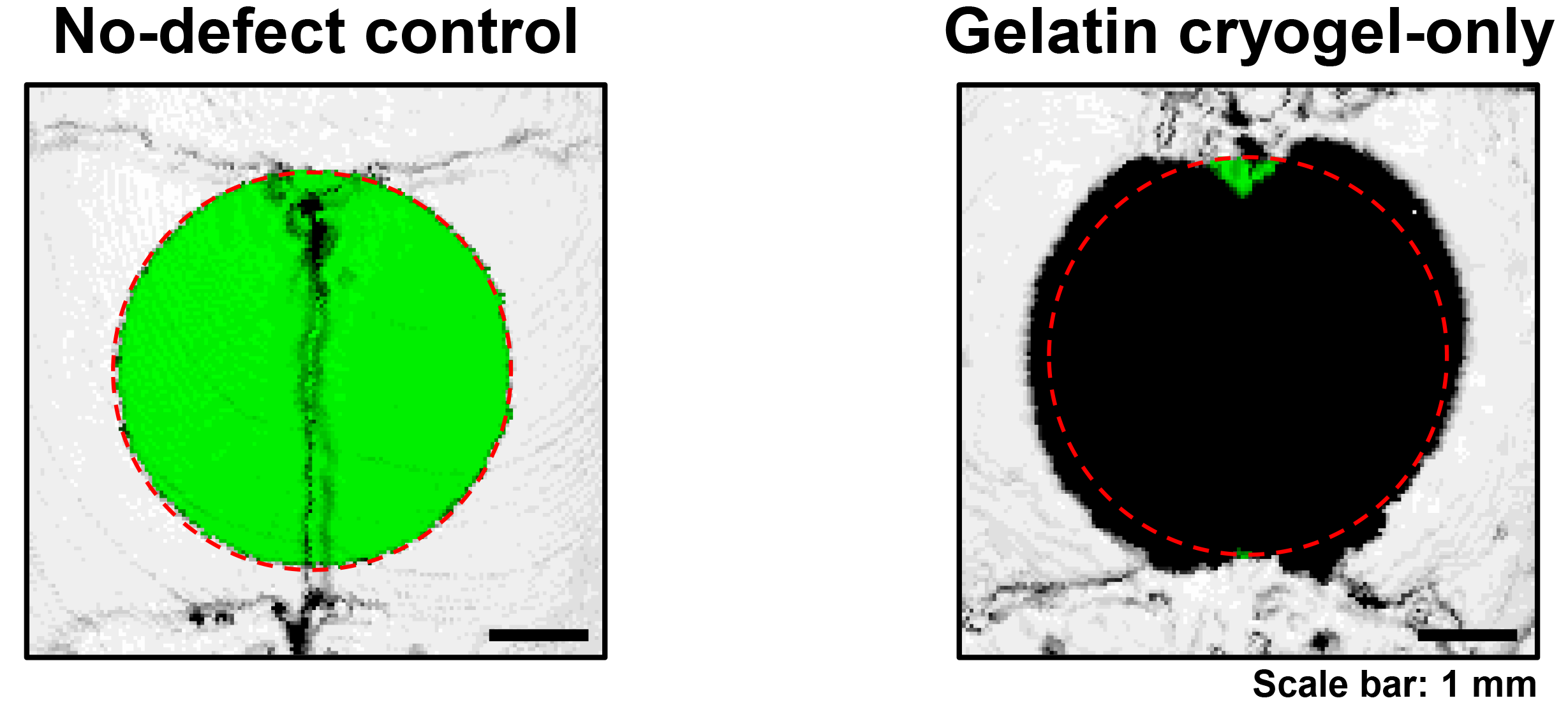


**Fig. S5.** Representative 3D micro-CT images of the no-defect control and gelatin cryogel-only groups at 8 weeks post-implantation. Green areas indicate bone tissues within the defined volume of interest.

**Movie S1.** AlphaFold2‑predicted 3D model of the Oct4‑30Kc19 fusion protein, displayed in continuous rotation about the X‑ or Y‑axes.

**Movie S2.** AlphaFold2‑predicted 3D model of the Cbfβ‑30Kc19 fusion protein, displayed in continuous rotation about the X‑ or Y‑axes.
